# Autoimmune GFAP astrocytopathy manifesting as sintilimab-induced myelitis: a Case Report and literature review

**DOI:** 10.3389/fimmu.2025.1595653

**Published:** 2025-08-27

**Authors:** Xue Ma, Xing Qin

**Affiliations:** Department of Neurology, The First Affiliated Hospital of Xi’an Jiao Tong University, Xi’an, China

**Keywords:** autoimmune glial fibrillary acidic protein astrocytopathy, myelitis, immune checkpoint inhibitors, sintilimab, glucocorticoids

## Abstract

Immune checkpoint inhibitors (ICIs) targeting programmed cell death protein 1 (PD-1), programmed cell death protein 1 ligand, and cytotoxic T-lymphocyte-associated protein 4, are important therapeutic approaches for malignancies. However, these novel treatment measures are associated with immune-related adverse events. We report the first reported case of autoimmune GFAP astrocytopathy-associated myelitis in a patient with hepatocellular carcinoma that was treated with sintilimab (an anti-PD-1 monoclonal antibody) immunotherapy. Additionally, literature review identified 21 previously reported cases of PD-1 inhibitor-associated myelitis, demonstrating similar clinical features. All patients received ICI discontinuation and high-dose glucocorticoid therapy, with the addition of other immune therapies in 15 patients. Clinical improvement was observed in 13 patients. Clinicians should consider autoimmune GFAP astrocytopathy-associated myelitis as a potential differential diagnosis among patients exhibiting neurological symptoms during or following ICI therapy.

## 1 Introduction

Immune checkpoint inhibitors (ICIs), which specifically target the programmed cell death protein 1/programmed death-ligand 1 (PD-1/PD-L1) axis and cytotoxic T-lymphocyte-associated protein 4, have great clinical efficacy as novel therapeutic agents in contemporary oncology practice. Sintilimab, a selective antibody against PD-1, inhibits the interaction between PD-1 and its ligands and restores anti-tumor immune responses (1). Sintilimab in combination with anti-angiogenesis agents has demonstrated significant clinical benefits in Chinese population with hepatocellular carcinoma (HCC) (2–5). Despite these advances, ICI-mediated immune hyperactivation may trigger diverse immune-related adverse events (irAEs) (6–8), including exacerbation or production of autoimmune neurological disorders (9), such as myasthenia gravis (10), motor polyradiculopathy (11), transvers myelitis (12, 13), and Guillain-Barr´e syndrome (14).

Autoimmune glial fibrillary acidic protein (GFAP) astrocytopathy is a central nervous system (CNS) inflammatory entity characterized by acute meningoencephalomyelitis with pathognomonic linear perivascular gadolinium enhancement on spinal or brain MRI and cerebrospinal fluid (CSF) lymphocytic pleocytosis, elevated protein, and GFAP-IgG positivity (15). Pembrolizumab-associated GFAP meningoencephalomyelitis was reported in a patient with non-small cell lung cancer (NSCLC) (16). Current understanding of ICI-induced GFAP astrocytopathy remains limited.

Herein, we describe the first case of sintilimab-induced GFAP astrocytopathy presenting as isolated myelitis in an HCC patient. And we conducted a literature review of all published cases of PD-1 inhibitor-associated myelitis, analyzing clinical phenotypes, therapeutic strategies, and prognostic outcomes. Our findings highlight the need for heightened clinical suspicion of autoimmune GFAP astrocytopathy in patients developing neurological manifestations during PD-1/PD-L1 blockade.

## 2 Case presentation

A 40-year-old male presented to our department with a 20-day history of gait disturbance. Neurological examination revealed mild instability on bilateral heel-to-shin testing and instability during the Romberg test with eyes closed. Muscle strength in all limbs, deep tendon reflexes, and superficial sensation were normal.

The patient had a 20-year history of hepatitis B virus (HBV) infection, treated with irregular Chinese herbal medicine for 19 years followed by 1 year of entecavir antiviral therapy. He was diagnosed with hepatocellular carcinoma (HCC, AJCC stage IIIa, T4N0M0) on February 1, 2024. The patient underwent a structured therapeutic regimen beginning with hepatic arterial infusion chemotherapy (HAIC) on February 5, 2024, using idarubicin, oxaliplatin, and fluorouracil. This was followed three days later by combined immunotherapy with sintilimab (200 mg) and bevacizumab (500 mg) on February 8, 2024. The treatment cycle repeated on March 1, 2024 with a second HAIC session using the same triple-agent protocol, subsequently reinforced by another dose of sintilimab and bevacizumab on March 5, 2024. A modified HAIC approach was implemented on April 12, 2024, omitting idarubicin while maintaining oxaliplatin and fluorouracil, followed by the final scheduled dual immunotherapy administration on April 15, 2024. Throughout this 10-week period, the patient received three cycles of HAIC (two with idarubicin-containing regimens) and three doses of combined ICI therapy at standardized intervals.

Laboratory findings revealed chronic hepatitis B infection with HBsAg >250.00 IU/L (normal <0.05), anti-HBe antibodies (0.14 S/CO, positive >1), and anti-HBc antibodies (7.04 S/CO, positive >1). Liver function abnormalities included elevated aspartate aminotransferase (57 U/L, normal 15-40), alkaline phosphatase (212 U/L, normal 45-125), gamma-glutamyl transferase (343 U/L, normal 10-60), direct bilirubin (5.3 μmol/L, normal 0-3.4), hypoalbuminemia (32.1 g/L, normal 40-55), and total bile acids (17.5 μmol/L, normal 3.4-17.1). High-sensitivity HBV DNA was undetectable. Blood electrolyte examination showed hypokalemia (2.86 mmol/L, normal 3.5-5.3), hypernatremia (148.7 mmol/L, normal 137-147), and hyperchloremia (112.4 mmol/L, normal 99-110). Hyperammonemia (68 μmol/L, normal 9-30) and elevated alpha-fetoprotein (15 ng/mL, normal 0-7) were noted. Erythrocyte sedimentation rate was slightly increased (20 mm/h, normal 0-15). His renal function and serum B-vitamin/micronutrient levels were normal.

**Figure 1:**
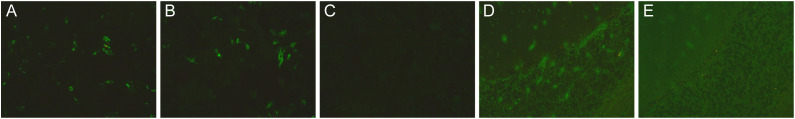
Serum and CSF and anti-GFAP antibodies of the patient detected by cell-based assay and tissue-base assay. Indirect immunofluorescence staining showed positive CSF anti-GFAP-ϵ antibodies at a titer of 1:3.2 **(A)**, anti-GFAP-α antibodies reactivity **(B)**, titer 1:1.1), and negative corresponding isotype controls **(C)** by cell-based assay. Representative images of CSF anti-GFAP antibodies positivity **(D)** and corresponding negative isotype controls **(E)** by tissue-base assay. Original magnification: ×200.

CSF analysis demonstrated pleocytosis (47 ×106/L, normal 0-8×106/L) and elevated protein (0.92 g/L, normal 0.12-0.6). Anti-GFAP-ϵ antibodies positivity at a titer of 1:3.2 and anti-GFAP-α antibodies reactivity (titer 1:1.1) in CSF were identified by cell-based assay (**Figures 1A–C**). CSF anti-GFAP antibodies positivity was further confirmed by tissue-based assay (**Figures 1D, E**). Serum anti-GFAP antibodies were negative. Both CSF and serum samples were negative for anti-aquaporin 4-IgG, anti-myelin oligodendrocyte glycoprotein-IgG, and paraneoplastic antibodies, including anti-Hu, anti-Yo, anti-Ri, anti-Ma2, anti-CV2, and anti-amphiphysin. No oligoclonal bands were detected in CSF or serum.

**Figure 2:**
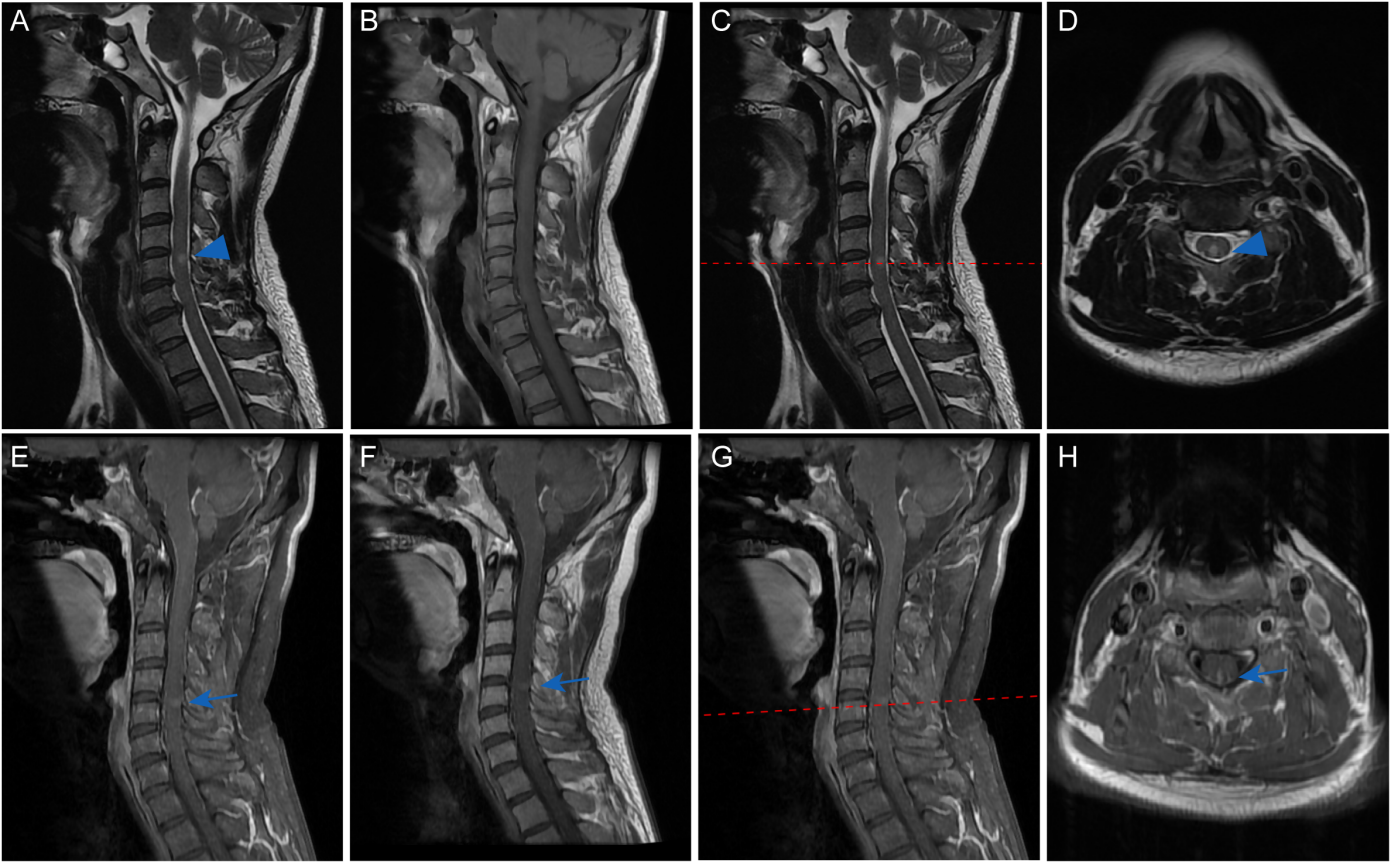
Cervical spinal MRI of the patient. Sagittal T2-weighted imaging demonstrated a discrete intramedullary hyperintensity spanning the C4-C5 levels (blue arrowhead, **(A)**, which mainly located in the posterior part on axial views (blue arrowhead, **(C, D)**. Corresponding T1-weighted sequences revealed iso-intensity without mass effect or cord expansion **(B)**. Contrast-enhanced sequences exhibited subtle linear enhancement along the posterior lesion margin on both sagittal (blue arrow, **(E-G)** and axial views (blue arrow, **H**). Mild leptomeningeal enhancement was noted at the cervicomedullary junction **(E-G)**.

Cervical spine MRI demonstrated T2-weighted hyperintensity at the C4–5 level, notably involving the posterior columns (**Figures 2A–D**). Gadolinium contrast-enhanced sequences revealed linear enhancement along the dorsal aspect of the lesion and mild leptomeningeal enhancement at the cervicomedullary junction (**Figures 2E–H**). Brain MRI and thoracolumbar spinal imaging showed no structural abnormalities. Therefore, the diagnosis of autoimmune GFAP astrocytopathy was made.

Given the patient’s history of chronic hepatitis B virus infection, high-dose intravenous methylprednisolone was deliberately avoided to prevent potential hepatic complications. Then the patient received a 3-day course of intravenous prednisone at 40 mg daily. Significant clinical improvement was observed within 5 days, with near-complete resolution of gait instability. The patient has not undergone any HCC-specific therapeutic interventions to date. Subsequent 9-month follow-up evaluations revealed normal liver function and serum AFP level. The patient sustained neurological remission at the last follow-up assessment.

## 3 Literature review

Through systematic literature review, we identified 21 documented cases of myelitis developing following PD-1 inhibitor therapy (12, 13, 16–24). Demographic characteristics, clinical features, and therapeutic outcomes treatment were summarized in **Table 1**. The median onset age of 59 years (range: 16-75) with male predominance (14/21, 66.7%). Malignancies predominantly included non-small cell lung cancer (10/21, 47.6%) and melanoma (7/21, 33.3%). Nivolumab (10/21, 47.6%) and pembrolizumab (12/21, 57.1%) were the most frequently administered PD-1 inhibitors. CSF analysis revealed median pleocytosis of 43.5 cells/μL (range 3-1195) and elevated protein levels (median 0.9 g/L, range 0.32-3.81). Five patients had positive autoantibodies: two serum anti–aquaporin-4 antibodies positive cases (22, 23), one CSF anti-GFAP antibodies positive case (16), and two CSF atypical antibody reactivity cases (16). Spinal MRI demonstrated predominant cervicothoracic involvement, with 95.5% (21/22) exhibiting thoracic segment abnormalities and 59.1% (13/22) showing cervical cord lesions. All patients underwent ICI discontinuation combined with immunosuppressive therapy: high-dose glucocorticoids monotherapy (6/21, 28.6%) or glucocorticoids in combination other immunotherapies (15/21, 71.4%). Most patients were responsive well to glucocorticoids treatment (14/21, 66.7%). Myelitis relapse occurred in 31.8% (7/21) of patients, requiring escalated immunotherapies, including IV immunoglobulin (n=1) (12), plasma exchange (n=1) (12), combination immunoglobulin and plasma exchange (n=1) (16), methylprednisolone with immunoglobulin (n=1) (12), and triple therapy with methylprednisolone, plasma exchange, and cyclophosphamide (n=1) (16). Clinical improvement was achieved in 63.6% (14/22) of patients. The overall mortality rate was 18.2% (4/22) with fatal outcomes attributed to sepsis (n=1) (16), respiratory failure (n=1) (13), or malignancy progression (n=1) (19).

**Table 1:** Findings of reported cases with myelitis-related adverse events secondary to PD-1 inhibitor therapy.

Items	All patients
Demographics	
Age at myelitis onset, median (range), years	59 (16-75)
Female	7 (33.3)
Malignancy	
NSCLC	10 (47.6)
Melanoma	7 (33.3)
Others^a^	4 (19.0)
ICI treatment	
Pembrolizumab	9 (42.9)
Nivolumab	8 (38.1)
Nivolumab and ipilimumab	3 (14.3)
Pembrolizumab, nivolumab, and ipilimumab	1 (4.8)
Clinical manifestation	
Myelitis	15 (71.4)
Myeloradiculitis	2 (9.5)
Encephalomyelitis	1 (4.5)
Encephalomyelitis and myeloradiculitis	2 (9.5)
Meningoencephalomyelitis	1 (4.5)
CSF findings	
Cells (n/μL), median (range) (n = 16)	43.5 (3-1,195)
Proteins (g/L), median (range) (n = 19)	0.9 (0.32-5.20)
Restricted OCB (n = 11)	4 (36.4)
Serum autoantibodies	
Anti-AQP4 antibodies	2/6
CSF autoantibodies	
Anti-GFAP antibodies	1/7
Spinal MRI involvement	
Cervical	13 (61.9)
Thoracic	20 (95.2)
Lumbar	3 (14.3)
Parenchymal enhancement on MRI	15/18 (83.3)
Treatment	
Glucocorticoids monotherapy	6 (28.6)
Glucocorticoids and other immune therapies^b^	15 (71.4)
Responsiveness to steroids	14 (66.7)
Relapse	7 (33.3)
mRS at onset, median (range)	4 (1-5)
mRS at last follow-up, median (range)	2 (0-6)
Outcome at last follow-up	
Clinical improvement	13 (61.9)
No improvement	4 (19.0)
Death	4 (19.0)
Follow-up from myelitis onset (months), median (range)	6 (2-30)

Data are n (%).

NSCLC, non-small cell lung cancer; CSF, cerebrospinal fluid; AQP4, aquaporin-4; MOG, myelin oligodendrocyte glycoprotein; MRI, magnetic resonance imaging; GFAP, glial fibrillary acidic protein; mRS, modified Rankin Scale.

a mesenteric inflammatory myofibroblastic tumor, classical Hodgkin lymphoma, clear cell renal carcinoma, and metastatic colorectal cancer.

b IV immunoglobulin, plasmapheresis, cyclophosphamide, tocilizumab, ruxolitinib, natalizumab, and infliximab.

## 4 Discussion

To the best of our knowledge, this is the first documented case of autoimmune GFAP astrocytopathy developing as an irAE following sintilimab therapy in an HCC patient. This unique presentation expands the clinical spectrum of ICI-associated neurological complications.

Clinical manifestations of autoimmune GFAP astrocytopathy typically involve meningoencephalitis or meningoencephalomyelitis, with isolated myelitis being a rare presentation (15, 25, 26). Distinct from the longitudinally extensive transverse myelitis commonly described in ICI-associated myelitis (12, 13, 16, 19, 24, 27), our patient exhibited short-segment posterior column involvement with a characteristic linear perivascular enhancement pattern. Notably, while radiation-related myelitis has been reported in some ICI-treated patients (12, 16, 18, 19), our case lacked prior radiotherapy exposure.

Diagnostically, our case fulfilled current criteria through CSF GFAP-IgG positivity confirmed via cell-based assay and tissue-based assay, a finding associated with higher diagnostic specificity compared to serum testing (15). The pleocytosis (47 ×106/L) and elevated protein (0.92 g/L) in CSF of our patient were in accordance with characteristic inflammatory CSF changes in autoimmune GFAP astrocytopathy (16, 25).

A previously reported nivolumab-treated NSCLC patient with GFAP meningoencephalitis demonstrated limited response to high-dose corticosteroids and plasma exchange, ultimately requiring natalizumab rescue therapy (16). This contrasts with our patient’s favorable steroid responsiveness, aligning with the typical corticosteroid-sensitive nature of GFAP astrocytopathy (15). Such therapeutic responses heterogeneity underscores the need for personalized treatment algorithms in ICI-associated autoimmune GFAP astrocytopathy.

Our systematic review reveals universal first-line high-dose glucocorticoids administration across reported cases, consistent with current guideline recommendations (28). However, most patients required additional immunomodulation due to incomplete functional recovery. Seven patients suffered from early relapses within several weeks due to rapid steroids tapering or glucocorticoids monotherapy inadequacy (12, 13, 16, 29). This clinical pattern suggests that patients with extensive myelitis lesions may benefit from aggressive initial immunotherapy regimens. Current evidence supports duration of the first steroid treatment should be continued for at least 3 months to substantiate recovery and prevent early relapses (6). Emerging biological agents including natalizumab, tocilizumab, and ruxolitinib show promise in refractory cases (16, 30), potentially offering targeted immunomodulation without compromising ICI anti-tumor activity.

Autoimmune GFAP astrocytopathy-associated myelitis represents a rare but potentially devastating neurological irAE with generally favorable steroid responsiveness. However, the suboptimal functional outcomes observed in longitudinally extensive myelitis cases necessitate early implementation of intensive, prolonged immunotherapy protocols. Future studies should focus on optimizing risk-stratified treatment algorithms and investigating targeted biological therapies to improve neurological outcomes while preserving anti-tumor immunity.

## Data Availability

The raw data supporting the conclusions of this article will be made available by the authors, without undue reservation.
